# Suicide by Firearm and Hormonal Imbalances: A Forensic Case Report With Literature Review

**DOI:** 10.7759/cureus.79803

**Published:** 2025-02-27

**Authors:** Saverio Gualtieri, Matteo Antonio Sacco, Maria Cristina Verrina, Chara Spiliopoulou, Isabella Aquila

**Affiliations:** 1 Department of Medical and Surgical Sciences, Magna Graecia University, Catanzaro, ITA; 2 Department of Forensic Medicine and Toxicology, School of Medicine, National and Kapodistrian University of Athens, Athens, GRC; 3 Institute of Legal Medicine, Department of Medical and Surgical Sciences, Magna Graecia University, Catanzaro, ITA

**Keywords:** forensic investigation, hormonal imbalance, psychiatric disorders, suicide, uterine fibroids

## Abstract

Hormonal imbalances play a crucial role in the development of various diseases, with a significant impact on the gynecological system, especially in the onset of uterine fibroids (UFs). UFs, also known as myomas or leiomyomas, are noncancerous uterine growths commonly observed in women, particularly during their reproductive years. By age 50, many women experience fibroids, though the condition's prevalence varies significantly across different ethnic groups. While most fibroids remain asymptomatic, those that are symptomatic can lead to several issues, such as irregular bleeding, pelvic discomfort, and complications during pregnancy.

Receiving a diagnosis of fibroids can be distressing, particularly when concerns arise about their potential link to more serious conditions. This distress can adversely affect a woman's quality of life and mental health, sometimes contributing to emotional distress, depressive symptoms, or even suicidal ideation. The management of UFs encompasses a spectrum of approaches, from careful monitoring to medical or surgical interventions, which are chosen based on the severity of symptoms and the patient’s preferences. Psychological support can be valuable in helping women manage the anxiety and fears associated with the diagnosis and treatment process. Consequently, the impact of UFs on women's mental health warrants attention. This paper aims to review the existing scientific literature on the potential link between UFs, hormonal imbalances, and an increased risk of psychiatric disorders.

## Introduction

Prevalence, risk factors, and management of uterine fibroids

Uterine fibroids (UFs) (leiomyomas) are benign tumors that originate from the smooth muscle cells of the uterus and are commonly associated with abnormal uterine bleeding, pelvic pain, and reproductive complications. While they can remain asymptomatic, in some cases, they contribute to urinary or intestinal issues, infertility, or pregnancy-related complications. Diagnosis is typically based on clinical examination, ultrasound, or imaging techniques, with the most frequent manifestations including irregular bleeding, pelvic masses, and obstetric complications [1].

The development of UFs is influenced by various risk factors, including female gender, advancing age, premenopausal status, and family history. Lifestyle and environmental factors, such as diet, obesity, vitamin D deficiency, and exposure to endocrine-disrupting chemicals, may also play a role. Despite advances in research, noninvasive long-term treatment options remain limited, and management depends on symptom severity, reproductive goals, and patient preferences. Treatments range from hormonal therapies and antifibrinolytic agents to surgical procedures like hysterectomy or myomectomy. The prevalence of fibroids increases with age, particularly among African American women who have a higher incidence compared to Caucasian women [2-4].

Psychological impact and potential links to mental health disorders

Several studies have reported a high prevalence of anxiety and depression among women with UFs, particularly those experiencing chronic pain, heavy menstrual bleeding, or fertility concerns. Hormonal imbalances have been suggested as a potential contributing factor to mood disturbances, with research indicating that women with symptomatic fibroids are more likely to experience depressive symptoms. While anxiety and depression are common in various chronic conditions, the association between fibroids and psychiatric disorders remains underexplored. Gynecologists play a crucial role in early screening and psychological support, especially for patients considering surgical interventions [5-8].

This paper presents a forensic case report and a literature review exploring the potential link between hormonal imbalances and psychiatric disorders, including suicidal behavior.

## Case presentation

This report concerns a 43-year-old woman who was found deceased in her home from a gunshot wound to the head. A firearm (Beretta 92 caliber 9X19 parabellum) was discovered in her right hand. A cartridge case was located on the mattress near her head, while a deformed bullet was recovered beneath the bed. The bed where the body was found had a blood-stained duvet.

The preliminary external examination revealed a gunshot wound with an irregular stellate appearance in the right temporal region, accompanied by visible burn marks. An analysis of both hands showed bloodstains only on the dorsum of the right hand. Thanatochronological parameters were assessed to estimate the time since death.

**Figure 1:**
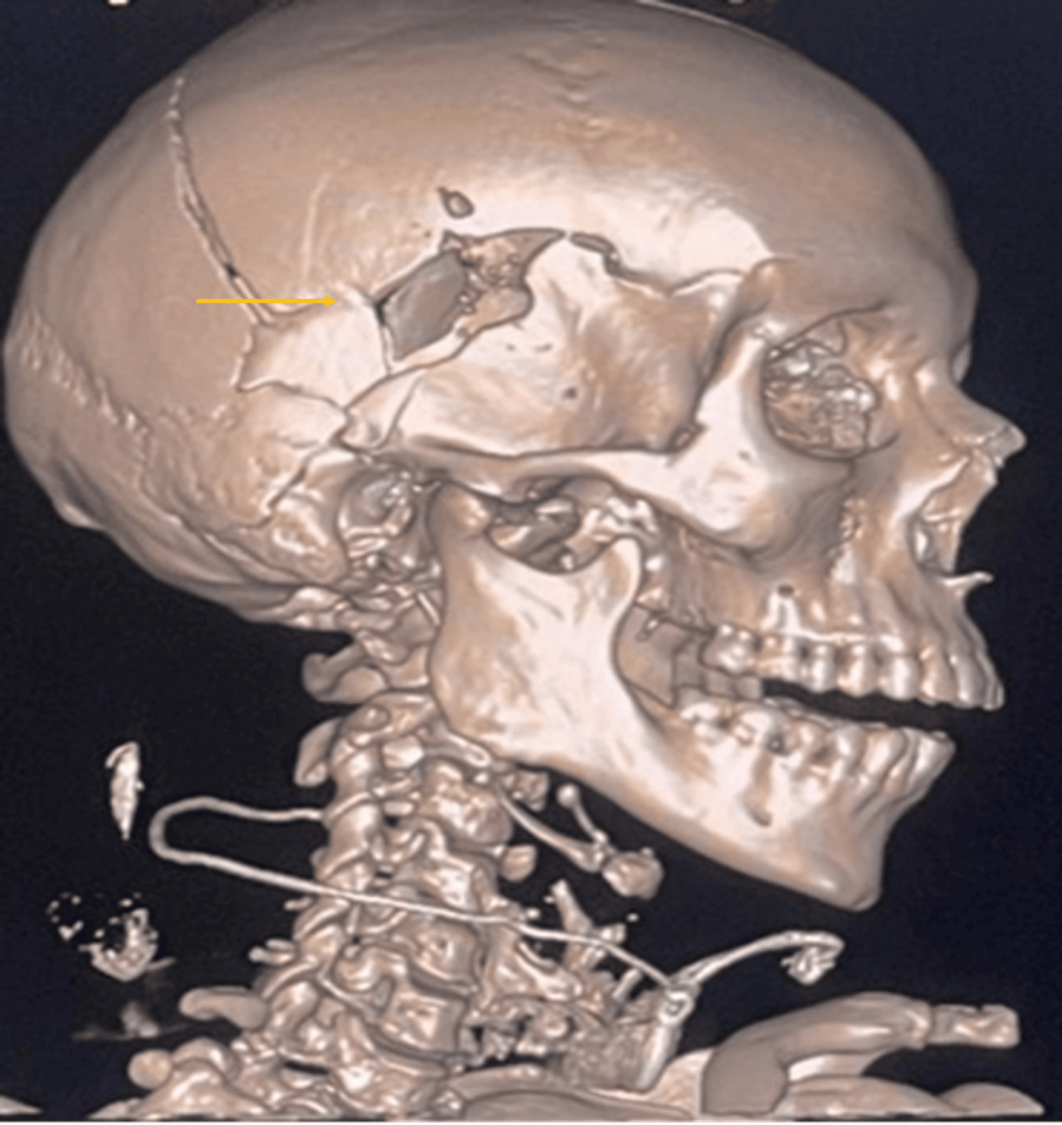
CT image of the cranial trauma showing a gunshot entry wound in the right temporal region

**Figure 2:**
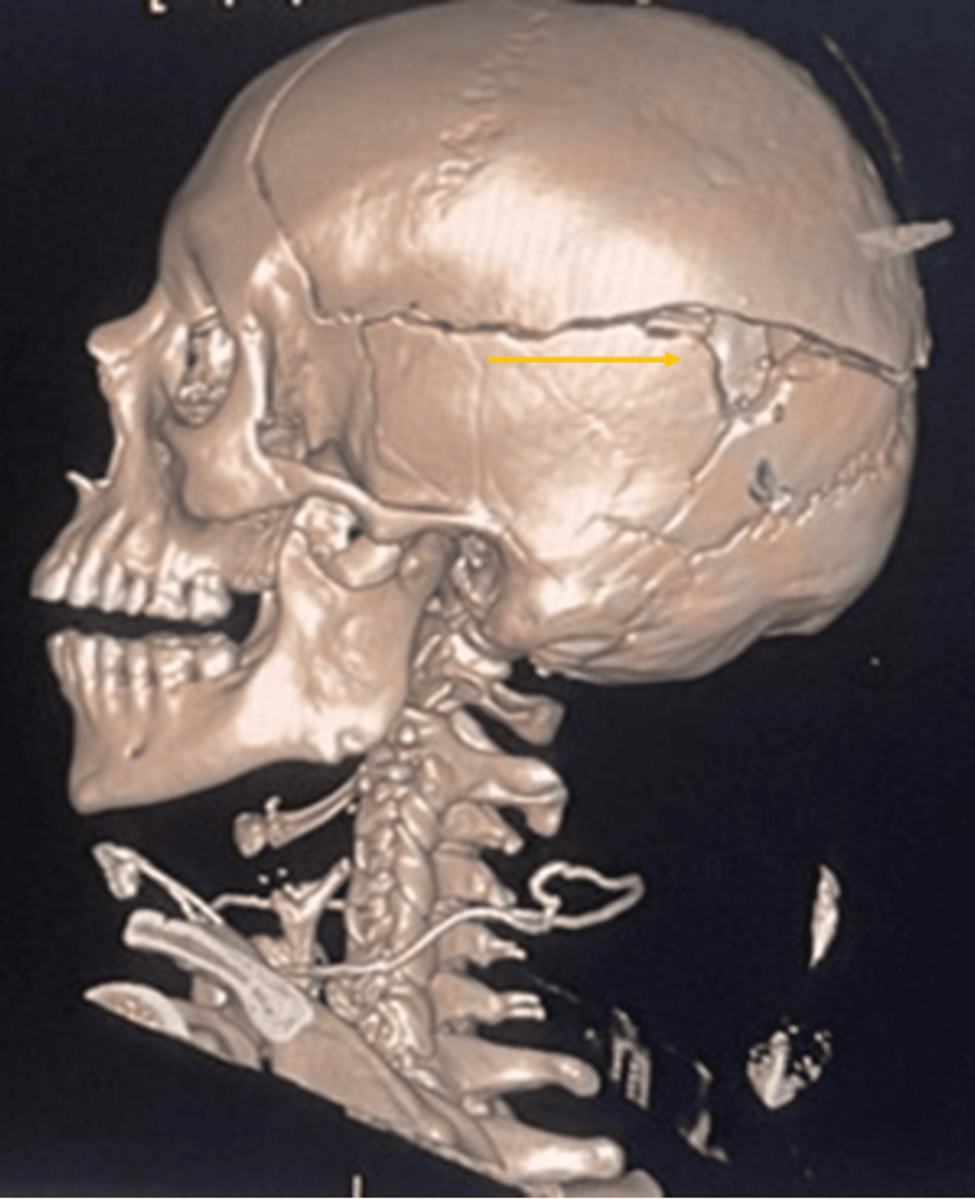
CT image of the cranial trauma showing a gunshot exit wound in the left parieto-temporal region. The image highlights adjacent bone fractures consistent with a high-energy firearm injury

During the scene inspection, a blister pack of Klaira (an oral contraceptive (OC)) was found at the victim's residence. A full-body CT scan confirmed the trauma dynamics and provided detailed visualization of the UFs, including their size and location. The internal examination involved a comprehensive evaluation of all organs and identified an intracranial injury consistent with the gunshot wound (Figure 1 and Figure 2).

**Figure 3:**
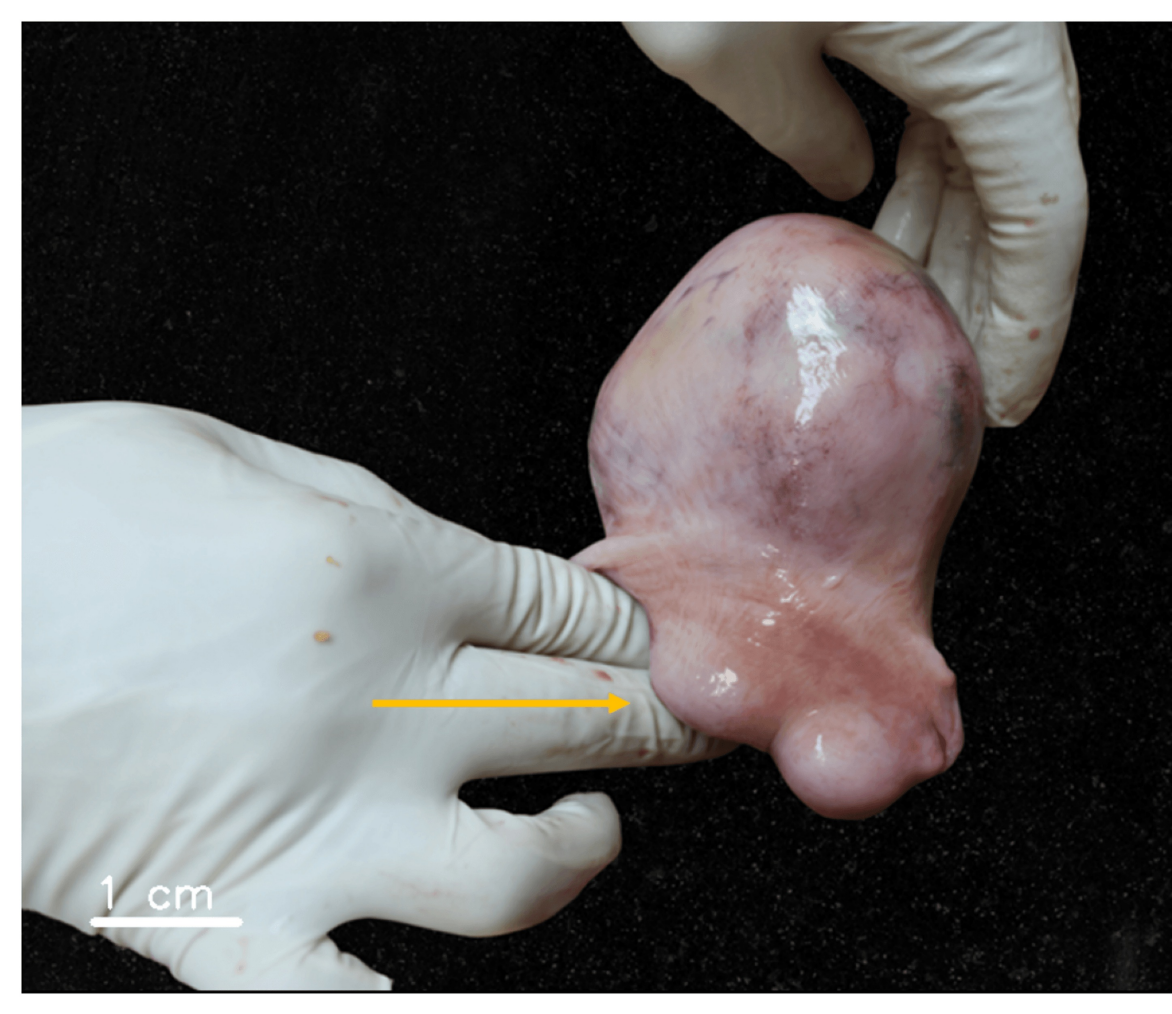
Postmortem examination of the uterus revealing multiple uterine fibroids measuring 2.5 cm

In the pelvic cavity, multiple uterine neoformations were observed. Two circular formations, measuring 2.5 cm, were located in the anterior region. Posteriorly, two smaller neoformations, each 0.5 cm in size, were identified. Additionally, a large neoformation with a woody consistency and spherical shape, measuring 7 cm in diameter, was detected in the upper uterine region (Figure 3).

Subsequent analysis of the woman’s clinical records focused on previous psychiatric consultations, as well as her family and work environments. No evidence of prior psychiatric disorders, history of self-harm, or other predisposing mental health conditions was found. However, suicidal behavior is multifactorial, and additional contributing factors such as psychosocial stress, personal crises, and underlying medical conditions were considered. While stressors in the workplace were noted, no major life events, substance abuse, or other known suicide risk factors were identified.

This case raised the question of whether hormonal imbalances could have played a role in exacerbating mood disturbances and increasing vulnerability to psychiatric disorders, including suicidal behavior. Studies suggest that chronic pain, hormonal dysregulation, and contraceptive use may influence emotional well-being, particularly in predisposed individuals [6-8]. Given the absence of a clear psychiatric history, a thorough review of the literature was conducted to explore whether hormonal fluctuations linked to UFs and contraceptive therapy could have contributed to the psychological distress observed in this case.

Materials and methods

This study was conducted in accordance with institutional forensic guidelines. A forensic investigation was conducted with a judicial inspection of the scene to reconstruct the sequence of events and perform initial examinations of the deceased. A preliminary external examination of the body was performed, focusing on thanatochronological parameters to estimate the time of death. Radiological analysis was carried out through a full-body CT scan using a Toshiba Aquilion CX64 scanner (Canon Medical Systems Corporation, Otawara, Tochigi, Japan), offering a spatial resolution of 0.35 mm. An internal examination was conducted to assess all internal organs in detail and identify any injuries. Additionally, a study of terminal ballistics was performed.

A psychological autopsy was conducted to complement the other examinations, aiming to reconstruct the victim's life experiences and identify personality characteristics, lifestyle, sociocultural context, and social relationships. The location of the incident, where both the suicide and the discovery of the body occurred (the victim’s home), was carefully documented. The circumstances surrounding the event were thoroughly analyzed. The methodology included structured interviews with family members to gather information on personal relationships, medical history, and potential psychiatric symptoms. Additionally, available personal communications, such as messages, emails, and written notes, were reviewed to identify possible indicators of emotional distress. Workplace conflicts were noted as a relevant stressor; however, due to limitations in available data, no formal interviews with colleagues were conducted. To evaluate workplace stressors, information was derived from family testimony regarding the victim’s professional environment, workload, and any known conflicts with colleagues or supervisors.

To ensure a systematic review of the existing literature, we conducted a search using PubMed, Scopus, and WOS databases. The search terms used were "uterine fibroids AND (depression OR anxiety OR suicide)". 

The inclusion criteria for the studies considered in this review required that they be published in peer-reviewed journals within the last 10 years and focus on the relationship between UFs and mental health disorders such as depression, anxiety, suicidal behavior, and psychiatric distress. Only studies involving human subjects with clinically diagnosed UFs were included. The exclusion criteria involved removing non-English publications, conference abstracts without full-text availability, and studies that focused exclusively on the treatment outcomes of fibroids without assessing mental health parameters. Finally, studies with insufficient statistical analysis or lacking clear methodological rigor were not considered for inclusion in the review.

**Figure 4:**
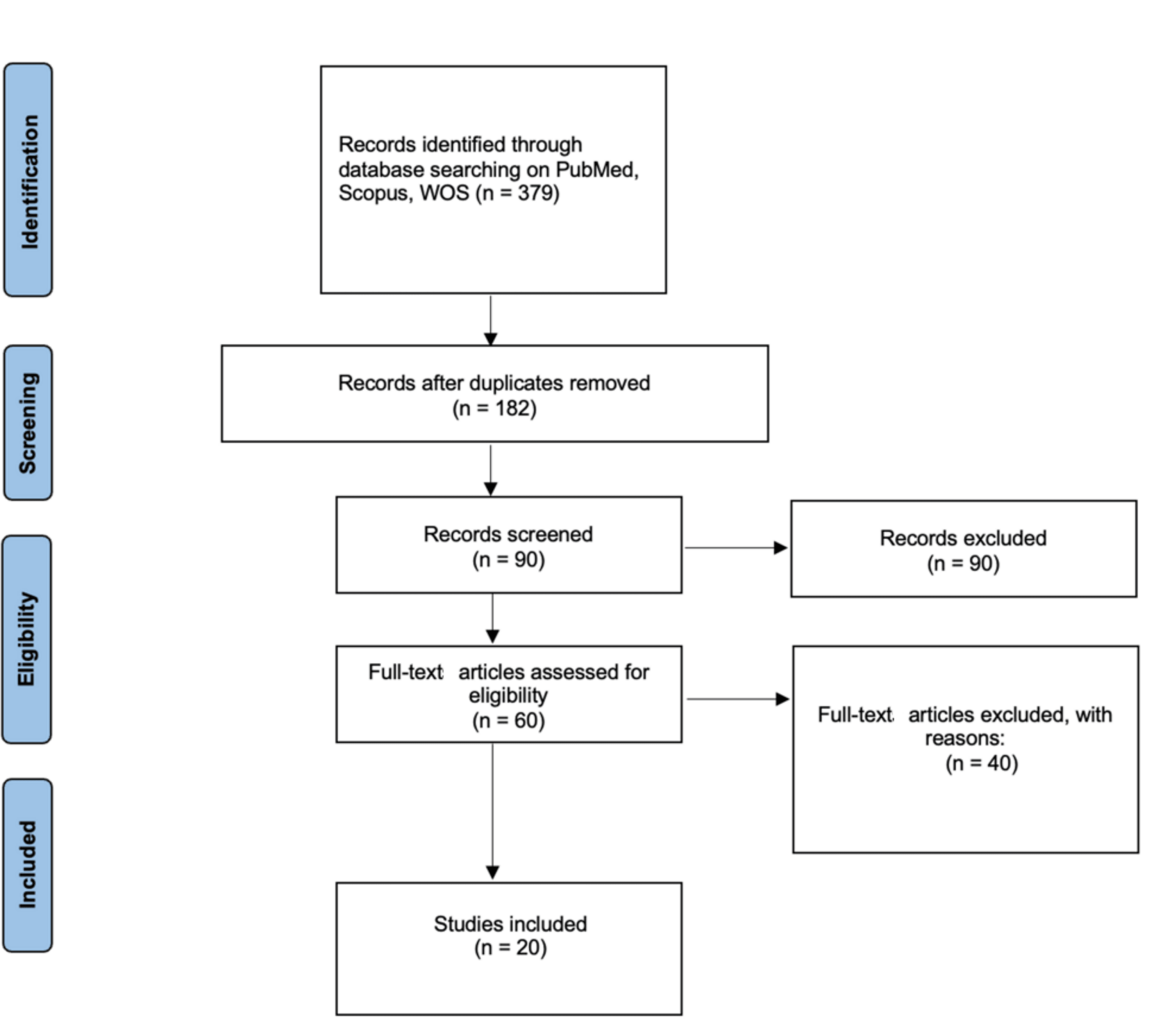
Results of the systematic literature review

A total of 379 studies were initially identified across all databases. After abstract screening and full-text assessment, 20 articles met the inclusion criteria and were incorporated into the review, after removing duplicates, irrelevant articles, and those lacking full text or strong relevance (Figure 4).

## Discussion

The link between UFs, hormonal imbalances, and mental health

The central question was whether UFs and hormonal imbalances could influence emotional states, potentially contributing to anxiety and depression. The literature review confirmed that UFs contribute to hormonal dysregulation and vitamin D deficiency, which have been linked to depressive symptoms [4]. Chronic pain and depression share several common biological pathways, including changes in neuroplasticity, monoamine neurotransmitters, brain-derived neurotrophic factors (BDNFs), and the glutamate system, including its receptor subtypes. Additionally, high rates of anxiety and depression are observed in patients suffering from chronic pain conditions, such as chronic pelvic pain, low back pain, and migraines [5-8].

In the present case, the victim had been using OCs daily for several months, according to records found at her residence. However, no direct medical records were available to confirm the exact duration of OC use. It is known that these medications can be associated with an increased risk of depressive symptoms, particularly during the first two years of use [5-20]. Nevertheless, the relationship between OC use and mood disorders remains complex, as individual biological susceptibility, psychological factors, and concurrent stressors can also influence emotional well-being.

Regarding her gynecological history, there was no prior medical documentation indicating the date of UF diagnosis. However, the postmortem examination revealed multiple fibroid formations, suggesting a long-standing condition. The absence of documented clinical history makes it challenging to determine whether the fibroids had been previously symptomatic or diagnosed. Given that UFs and mental health disorders are chronic conditions that often experience delays in diagnosis, establishing the exact timing of disease onset is difficult. Evidence suggests that the relationship between UFs and mental health disorders may be bidirectional, with a shared underlying cause such as hormonal imbalances. However, the role of hormonal fluctuations in psychiatric symptoms remains an area for further investigation.

Obstetricians and gynecologists have a critical role in the early detection, prevention, and management of mood disorders through routine screening for psychological symptoms. When counseling patients about treatment options, especially more invasive procedures like hysterectomy, it is crucial to consider the potential impact on mental health.

Several biological mechanisms may explain the potential connection between UFs and mood disorders. One plausible explanation is that hormonal dysregulation could be a common factor in the development of both conditions. The review highlighted that sex steroid hormones play a role in fibroid pathogenesis and that serum hormone levels differ between women with depression and those without. Psychological stressors like depression can disrupt the hypothalamic-pituitary-adrenal (HPA) axis, which, in turn, may alter the hypothalamic-pituitary-ovarian axis and affect the production of sex steroid hormones [8,9].

In the literature review, emerging evidence suggests a bidirectional association between UFs and mental health disorders, particularly depression and anxiety. Cheng et al. analyzed a group of 50 patients diagnosed with fibroids and compared them to 50 healthy individuals [9]. They measured serum estradiol levels using a chemiluminescent immunoassay and assessed anxiety and depression through standardized self-rating scales (Self-Rating Depression Scale (SDS) and Self-Rating Anxiety Scale (SAS)) [9]. The results showed that women with UFs had significantly higher anxiety and depression levels compared to the control group. Han et al. investigated the causal relationship between psychological distress and UFs using Mendelian randomization (MR) [7]. Analyzing genetic variants (single-nucleotide polymorphisms, SNPs) associated with depression, anxiety, mood swings, and panic attacks, researchers found that genetic predisposition to depressive symptoms (OR = 1.563, p = 0.001) and major depressive disorder (MDD) (OR = 1.176, p = 0.007) significantly increased the risk of UFs. Mood swings also showed a weaker association (OR = 1.578, p = 0.024) [7,10]. 

Chiuve et al. examined the relationship between UFs and mental health disorders, specifically depression, anxiety, and self-directed violence [3]. Using a large insurance claims database, researchers analyzed data from 313,754 women with UF and compared them to 627,539 women without UF, adjusting for demographics and comorbidities [3].

The findings revealed that women with UF had a higher risk of developing depression (HR = 1.12), anxiety (HR = 1.12), and self-directed violence (HR = 1.46) compared to those without UF [11]. The risk was even higher among women who experienced pain and heavy menstrual bleeding, with HRs increasing to 1.21 for depression, 1.18 for anxiety, and 1.68 for self-directed violence. Additionally, women who underwent a hysterectomy had an even greater likelihood of mental health issues, with HRs of 1.22 for depression, 1.13 for anxiety, and 1.86 for self-directed violence [3].

These results suggest a link between UF and mental health conditions, particularly in cases involving severe symptoms or surgical intervention. The study highlights the need for comprehensive care that considers both physical and psychological well-being in women diagnosed with UF [12-20].

The analyzed studies collectively suggest a potential association between UFs and mental health disorders, particularly depression, anxiety, and self-directed violence. Evidence indicates that hormonal imbalances, chronic pain, and heavy menstrual bleeding may contribute to psychological distress in affected women. Additionally, genetic predisposition to MDD appears to increase the risk of developing fibroids, while surgical interventions like hysterectomy further elevate the likelihood of mental health issues. These findings emphasize the importance of a multidisciplinary approach in managing UFs, integrating both gynecological and psychological care to improve patient well-being.

Potential contributing factors to psychological distress in the case presented

In the case presented, there was no medical record indicating that the woman had been previously diagnosed with UFs. However, she was found to be using OCs, which were recovered at the scene. According to available records, she had been taking OCs for several months, but the exact medical reason for their prescription remains unclear. OCs can be prescribed for various gynecological conditions, including menstrual irregularities, endometriosis, and contraception, but no documented diagnosis of fibroids was found in her clinical history.

Regarding her personal and family background, there was no reported history of psychiatric disorders among her immediate relatives. She was single and had no children, and no significant obstetric complications were documented. Given the absence of prior psychiatric consultations and the lack of a formal fibroid diagnosis, this case raises the question of whether undiagnosed hormonal imbalances may have played a role in mood disturbances. However, without clinical records or biochemical evidence, this remains a theoretical hypothesis rather than a definitive conclusion.

Approximately 90% of suicides are associated with underlying mental disorders, with major depressive disorder being implicated in 60% of cases. Sociocultural factors also play a critical role in triggering self-harming behaviors. Suicides typically occur during periods of personal, family, or socioeconomic crisis [10]. The influence of hormones on mood regulation and behavior is well documented, particularly in how they affect an individual’s reaction to environmental stressors. Thus, while hormonal dysregulation may contribute to mood changes, it is not the sole determining factor, and it remains unclear whether it had a direct impact in this case.

In the reported case, no clinical diagnosis of psychiatric disorders or family history of mental illness was identified. However, the woman had experienced relational problems at work, which could have contributed to psychological distress. Workplace conflicts and chronic stress are well-known risk factors for depression and suicidal behavior, particularly in the absence of strong social support. Given the multifactorial nature of suicide, it is important to consider all possible contributing factors, including personal stressors, biological predispositions, and environmental influences.

The need for a multidisciplinary approach and future research directions

While work-related stress alone can be a significant trigger for suicide in some individuals, it is important to consider it alongside other potential contributing factors. In this case, hormonal imbalances, chronic pain from undiagnosed UFs, and the use of OCs (known to influence mood) could have contributed to her emotional state. However, the absence of direct hormonal assessments or psychiatric evaluations makes it impossible to determine whether hormonal factors played a significant role or whether psychosocial stressors were the primary drivers. The interaction of these elements may have contributed cumulatively to emotional distress, emphasizing the need for a multidisciplinary approach in forensic investigations that considers both biological and psychosocial factors.

A primary limitation of this study is the absence of postmortem hormonal analysis, which prevents the establishment of a biochemical correlation between hormonal imbalances, fibroids, and potential mood disturbances. Additionally, postmortem hormone testing poses technical challenges due to degradation processes that can alter hormone levels, making interpretation difficult without premortem baseline measurements. In forensic practice, hormonal assessments are not yet standard in psychological autopsies, which limits the ability to retrospectively explore endocrine influences on psychiatric vulnerability.

Future forensic investigations should consider standardized postmortem endocrine analysis to better understand the role of hormonal dysregulation in suicide cases. Prospective clinical studies are also necessary to establish biomarker-based evidence, helping to clarify the potential interactions between hormonal fluctuations, psychiatric disorders, and suicidality.

## Conclusions

This case presents a forensic investigation of a suicidal death by firearm. The victim had no documented history of psychiatric disorders, and workplace-related conflicts were identified as a significant stressor. Postmortem findings revealed the presence of UFs and ongoing use of OCs, which raised the question of whether hormonal imbalances may have contributed to psychological distress. However, in the absence of premortem psychiatric evaluations or biochemical assessments of hormone levels, this remains a speculative hypothesis rather than a definitive causal link.

Suicide is a complex and multifactorial event influenced by psychological, social, and biological factors. While research suggests that hormonal fluctuations may be associated with mood disturbances, other explanations, such as undiagnosed psychiatric conditions or cumulative psychosocial stressors, must also be considered. Given these complexities, this case underscores the importance of a multidisciplinary forensic approach that integrates psychiatric, endocrinological, and social perspectives when investigating potential risk factors for suicide. Further prospective studies examining hormonal biomarkers and mental health outcomes in similar cases are needed to provide a more definitive understanding of this relationship.
